# Outcome and reproducibility of data concerning the Oxford unicompartmental knee arthroplasty: A structured literature review including arthroplasty registry data

**DOI:** 10.3109/17453674.2011.566134

**Published:** 2011-04-05

**Authors:** Gerold Labek, Kathrin Sekyra, Wolfram Pawelka, Wolfgang Janda, Bernd Stöckl

**Affiliations:** Department of Orthopaedics, Innsbruck Medical University, Innsbruck, Austria

## Abstract

**Background and purpose:**

The reproducibility of results and potential confounders in sample-based studies is important to consider in the assessment of studies. Comprehensive arthroplasty registers could serve as a reference dataset for comparative analyses. We analyzed an implant that is frequently used worldwide, the Oxford unicompartmental knee replacement, in order to identify potential confounders inherent in the datasets and to evaluate the outcome achieved with this implant.

**Methods:**

We performed a structured literature review of the data published on the revision rate of the Oxford medial unicompartmental arthroplasty. Both clinical follow-up studies and worldwide registry data were included. Confidence intervals were calculated to determine the statistical significance of differences.

**Results:**

A substantial proportion of the published data (52–68% depending on the method of calculation) is derived from studies involving participation of the institution that developed the implant. The results published by this group show a statistically significant deviation from the reference datasets from registers or independent studies. Data from the developing hospital show mean revision rates that are 4 times lower than those based on worldwide register data, and 3 times lower than the ones quoted in independent studies. On average, the data published in independent studies are reproducible in registry data.

**Interpretation:**

A conventional meta-analysis of clinical studies is substantially affected by the influence of the developing hospital, and is therefore subject to bias. For assessment of the outcome of implants, registry data are superior and, in terms of reference data for the detection of potential bias factors in the literature, could make an essential contribution to meta-analyses.

Two main data sources are available for the assessment of the outcome of arthroplasty: sample-based clinical studies and national arthroplasty registries. Compared to clinical follow-up studies, registry data feature several essential differences (Graves 2010).

Clinical studies are mainly conducted in specialized centers that are not representative of the average orthopedic center in all aspects, for example, regarding the number of patients treated and, as a consequence, the training of staff and their personal expertise. Study design or patient selection may introduce further bias factors. Even publication bias can have a potentially relevant effect on the data published.

National arthroplasty registers, by contrast, include all surgeries performed in a country and can thus avoid or considerably reduce these bias factors. On the other hand, data from registries reflect the circumstances under which they were collected, such as surgical procedures or the respective public health system, and can thus have an impact on the outcome. Also, the evaluation procedures applied, such as designation of implant variants to cohorts, could possibly lead to misinterpretations (Labek et al. 2008).

The expectation that published results can be reproduced in their own practice is essential for the readers of scientific literature. This is equally true for the assessment of implants and decisions by health authorities, since published scientific findings are considered in a variety of decision-making processes. In general one should take into consideration that results presented in scientific literature would not be expected to be deterministic, but subject to random and sampling errors that can be calculated and described using confidence intervals.

One of the tasks of the EU Commission's EUPHORIC (European Public Health Outcome Research and Indicator Collection) project was to examine the extent to which published data are reproducible in an average situation. The objective was to identify potential bias factors and to develop a suitable methodology for this particular purpose. Data from complete and high-quality national registries were used as reference benchmark values reflecting the outcome in average patient service.

The Oxford unicompartmental prosthesis has been one of the most frequently used implants in the field of knee arthroplasty worldwide for many years, particularly for the isolated replacement of the medial compartment. We critically analyzed the outcome of the Oxford unicompartmental implant and the quality of the published literature dealing with this implant.

## Materials and methods

We conducted a web-based literature search using PubMed as a first step. This was followed by a manual literature search, and also a direct request for literature from the manufacturer of the implant.

The inclusion criteria for consideration in the subsequent evaluation were: unambiguous identification of the implant, revision rate data either presented in the text or unambiguously calculable from the data therein, English or German language publications in Medline-listed, peer-reviewed journals.

23 publications were identified and analyzed in full text (Goodfellow et al. 1988, Carr et al. 1993, Lewold et al. 1995, Murray et al. 1998, Vorlat et al. 2000, 2006, Svärd and Price 2001, Emerson and Higgins 2004, 2008, Jahromi et al. 2004, Lisowski et al. 2004, Rajasekhar et al. 2004, Langdown et al. 2005, Price et al. 2005 a,b, Verdonk et al. 2005, Pandit 2006, Vorlat et al. 2006, Berend et al. 2007, Kort et al. 2007a, b, Luscombe et al. 2007, Koskinen et al. 2007, 2008). 20 of these publications were monocenter studies and 3 of them were based on multicenter evaluations (Langdown et al. 2005, Price et al. 2005b. Vorlat et al. 2006).

Clinical follow-up studies were compared to datasets from arthroplasty registers. The analysis included journal publications as well as annual registry reports that were accessible via http://www.efort.org/getdoc/1b923b01-41d2-4587-bac2-7ca7a11e613e/Arthoplasty-Registers.aspx. 3 journal publications were available from arthroplasty registers in Finland and Sweden (Lewold et al. 1995, Koskinen et al. 2007, 2008). Annual reports were available from Australia (Annual Report 2008), Sweden (Annual Report 2007), and Finland (2006 Implant Yearbook). These allowed derivation of the values required for indicator calculation.

The main evaluation criterion was the indicator ‘revision rate’, a variation of which, ‘revisions per 100 observed component years’, was used for the comparative assessment. It was applied in accordance with the Australian National Arthroplasty Registry's definition (Australian National Joint Replacement Registry Annual Report 2008).

The basic idea of this parameter is to summarize all patients' individual years after surgery as ‘observed component years’, during which they are at risk of revision (no. of cases × average follow-up period), and to compare this value with the number of revisions observed in this cohort. This method of evaluation allows considering the number of cases and the follow-up period in any publication with respect to its impact on the average results. Larger studies and longer follow-up periods are given higher weight in the calculation due to the higher number of observed component years. This procedure enables direct comparison of different studies and data sources expressed in one value. A value of 1 revision per 100 observed component years corresponds to a revision rate of 5% at 5 years or a 10% revision rate at 10 years in conventional follow-up studies.

The journal publications were analyzed regarding the source of publication, authors, geographic region, number of cases, and follow-up period. Any publication indicating the Nuffield Orthopaedic Centre in Oxford and/or the Nuffield Department of Orthopaedics, Rheumatology and Musculoskeletal Sciences of the University of Oxford as the contact address and/or naming Prof. Goodfellow or Prof. Murray as authors or co-authors was rated as a ‘publication by the development team’.

For all data sources, all data were pooled in a standardized way. For each parameter, with the exception of follow-up times, exact values were required for inclusion in the study. If no specific follow-up times, but mere follow-up periods were given, a linear distribution of cases was assumed.

To determine statistical significance, 95% confidence intervals (CIs) were calculated. Confidence intervals were calculated using Circulator software version 4, an Excel-based program from the University of Adelaide. Further statistical evaluations were not performed owing to variability in the basic data and in the designs of the studies included.

## Results

20 of the 23 publications were conventional clinical follow-up studies (Goodfellow et al. 1988, Carr et al. 1993, Murray et al. 1998, Vorlat et al. 2000, Svärd and Price 2001, Emerson and Higgins 2004, 2008, Jahromi et al. 2004, Lisowski et al. 2004, Rajasekhar et al. 2004, Langdown et al. 2005, Verdonk et al. 2005, Price et al. 2005a, b, Pandit 2006, Vorlat et al. 2006, Berend et al. 2007, Price et al. 2005a, b, Price et al. 2005, 2007, Kort et al. 2007a, b, Luscombe et al. 2007), 7 of which came from the developing hospital in Oxford (Goodfellow et al. 1988, Carr et al. 1993, Murray et al. 1998, Langdown et al. 2005, Price et al. 2005a, b, Pandit 2006). 3 journal publications were based on data from national registers (Lewold et al. 1995, Koskinen et al. 2007, 2008).

The majority of these publications were of European origin.

### Length of follow-up

With an average follow-up of 10 years, the follow-up periods of the development team were double those referred to in the independent studies, where the average was 5 years. With a total of 1,559 patients, the cumulative number of cases in the publications from the developing hospital was slightly higher than the number of cases from independent users' clinics, comprising 1,445 patients. 52% of all cases were published by authors from the developing hospital.

### Studies from the inventor hospital compared to independent studies

Of the total population presented in clinical studies (comprising 3,004 cases), 155 had to undergo revision surgery. This corresponds to a proportion of revisions of 5.2%. With a value of 0.45 (CI: 0.35–0.57), the probability of reoperation according to the ‘revisions per 100 observed component years’ indicator was statistically significantly lower in the developing hospital than the values quoted in the independent studies, where the comparative value was 1.2 (CI: 0.99–1.5). The revision rate published in papers from the developing hospital was therefore 2.7 times lower than in independent studies, a difference that is statistically significant.

Summarizing the data, as is done in conventional meta-analyses, yielded a revision probability of 0.70 (CI: 0.60–0.82) revisions per 100 observed component years. This outcome differed statistically significantly from the average value published by the development team from Oxford, but not from that derived from independent publications. 3 journal publications were based on data from national registries in Sweden and Finland (Lewold et al. 1995, Koskinen et al. 2007, 2008) (Table 1).

**Table 1.** Description of basic data with respect to data source

	No. of publications	Follow-up period	Revisions/ primaries (%)	No. of primary cases	No. of revision cases	Observed component years	Revisions per 100 observed component years	CI
Inventor studies	7	9.6	4.3	1,559	67	15,029	0.45	0.35–0.57
Independent clinical studies	13	5.0	6.1	1,445	88	7,205	1.22	0.99–1.50
Total clinical studies	20	7.4	5.2	3,004	155	22,234	0.70	0.60–0.82
Registry-based journal publications	3	9.0	14.5	1,951	283	17,638	1.60	1.43–1.80
Annual registry reports	3	3.5	6.9	11,985	825	42,037	1.96	1.83–2.10

### Registry data

Analysis of the annual national arthroplasty registry reports of Australia, Finland, and Sweden revealed average outcomes ranging from 1.7 to 2.3 revisions per 100 observed component years, with Sweden achieving the best results. The differences showed a maximum factor of 1.2 though, and were not statistically significant (Table 2).

**Table 2.** Outcome of the Oxford unicompartmental knee arthroplasty, by country

	Follow-up period	Revisions/ primaries (%)	No. of primary cases	No. of revision cases	Observed component years	Revisions per 100 observed component years	CI	Factor difference to the average
Australian Registry	3.55	6.88	8,644	595	30,720	1.9	1.8–2.1	0.97
Finnish Registry	2.72	6.23	2,361	147	6,417	2.3	2.0–2.7	1.2
Swedish Registry	5	8.5	980	83	4,900	1.7	1.4–2.1	0.86
Total/Average	3.51	6.88	11,985	825	42,037	2.0	1.8–2.1	

Sources: Australia: Annual Report 2008; Finland: Yearbook 2006; Sweden: Annual Report 2007.

A comparison of annual reports and journal publications from registries showed considerably longer follow-up periods, with a smaller number of patients involved in the journal publications. Since journal publications are based on defined cohorts to examine specific topics such as long-term outcome, this result is not surprising. There were no statistically significant differences in revision rate.

The average deviations between individual register-based studies were also very low, and they were not statistically significant. The average value for all studies deviated from the average comprehensive, worldwide registry data by a factor of 1.2.

### Registry-based studies compared to studies from the developing hospital

At an average of 9 years, the follow-up periods for publications based on registry datasets were similar to those for the developing hospital. The number of cases included in registry publications was larger by a factor of 1.3, and was thus in a comparable range. At 1.6 (CI: 1.4–1.8) revisions per 100 observed component years, the revision probability shown in this dataset exceeded that quoted in the developers' publications by a factor of 3.6. This difference was statistically significant.

In the comprehensive datasets of arthroplasty registries, a value of 2 (CI: 1.8–2.1) revisions per 100 observed component years became apparent. Thus, the revision rate was 4.4 times higher than in the developers' publications and 2.7 times higher than in the average of all sample-based journal publications. The differences between the datasets of clinical studies by implant developers and pooled data were statistically significant, which was not, however, the case for the difference between inventor studies and independent studies.

### Registry-based studies compared to independent studies

The difference between inventor-independent studies and registry-based journal publications only amounted to a factor of 1.3, and to a factor of 1.6 in comparison with annual reports of national arthroplasty registries; comparison of the cumulative value of all clinical publications to the values recorded in national arthroplasty registry reports yielded a difference factor of 2.8. This higher value clearly reflects the influence of publications from the inventor hospital.

### General considerations

When assessing the clinical relevance of the differences detected, one should take into account the usual deviation of individual hospitals or datasets from the mean. The national registries of Sweden and Denmark publish such data. Here, the best national hospitals deviated from the mean by a factor of 2–3 at most (Danish Hip Arthroplasty Register Annual Report 2006, Swedish Hip Arthroplasty Register Annual Report 2007; Swedish Knee Register Annual Report 2009). Deviations within these limits can therefore be assumed to be plausible and to represent differences in revision rates that may be caused cumulatively, e.g. due to personal skills, in-house standards and quality assurance, or patient selection.

## Discussion

The average results on the implant published by the inventor team differed markedly from the outcome published in independent clinical studies or shown by worldwide national arthroplasty registry data. They also exceeded the maximum deviation of individual departments (due to factors such as personal expertise and patient selection) from the national mean that has been registered for hip and knee prostheses in countries such as Sweden and Denmark.

The cause of this divergence can only be an issue for speculation. Irrespective of the reasons for these deviations, however, the average surgeon should be aware of the fact that the outcome published by the inventing center appears to be hardly reproducible in average patient care and other institutions. Thus, the published results of this group are only of limited value for decision making by other users since they cannot expect to be able to reproduce such excellent results.

On average, the revision rate data of the Oxford team have been 2.7 times lower than the revision rates quoted in the independent literature, and 4.4 times lower than the results from worldwide registry data. This means that on average, publications involving revisions from this group only match 23% of the revisions documented in worldwide registers, and 37% of the revisions published in independent studies.

Even though one third of the clinical studies (7/22) have originated from the inventor group, these papers account for one half of all cases published worldwide. Owing to the longer follow-up periods, the value for observed component years reached 65%.

While multiple mentions of the same patients in different studies unfortunately cannot be excluded in a literature analysis, this does not affect the impact publications have on experts and decision-makers. As a rule, major studies covering longer periods of follow-up are assigned superior value.

The implant development team in Oxford has therefore been clearly overrepresented in the clinical literature, which—along with the discrepancy regarding clinical outcome—has had a statistically significant influence on all the published results. In a conventional analysis of the clinical literature, this influence therefore represents a confounder that could also affect assessment of the product by stakeholders.

By contrast, the independent clinical literature puts forward revision rates that are 1.6 times lower than the comparative figures in registries. These differences can, however, be plausibly explained by factors such as higher surgical expertise. In general, studies without participation of the developers of the implant can be said to have good reproducibility.

The variation in results is clearly less in registries of different countries than it is in the clinical literature. This applies to both annual reports and registry-based journal publications. Apart from the larger numbers of cases, it is probably the minimization of confounding factors, which basically cannot be excluded in sample-based studies, that accounts for this effect.

Moreover, the impact of a single group on the results is automatically limited by the wider scope of data collection. For the assessment of outcome results on orthopedic implants, registry data are therefore superior to clinical studies. The potential influence of national circumstances can be quantified and narrowed by comparing data from different countries.

Registry data can be used as a benchmark in the assessment of clinical studies, particularly when it comes to evaluation of whether relevant bias factors could possibly have an influence on the outcome. Thus, registry data can provide a valuable contribution to the assessment of outcome data.

Regardless of the confounding factors detected in the clinical literature, registry data on the Oxford unicompartmental knee prosthesis indicate similar performance of this implant in comparison with other well-performing products for unicompartmental knee arthroplasty.
